# From Design to Clinical Use: mRNA Vaccines for Infectious Diseases and Cancer

**DOI:** 10.3390/vaccines14030202

**Published:** 2026-02-25

**Authors:** Yulin Cui, Ziyue Liang, Hua Cong

**Affiliations:** Department of Pathogenic Biology, School of Basic Medicine, Cheeloo College of Medicine, Shandong University, Jinan 250012, China; 202316280@mail.sdu.edu.cn (Y.C.); 202416291@mail.sdu.edu.cn (Z.L.)

**Keywords:** mRNA vaccine, circular RNA vaccine, lipid nanoparticle (LNP), vaccine delivery system, infectious disease, cancer immunotherapy

## Abstract

mRNA vaccines represent a revolutionary advance in vaccinology, boasting advantages like rapid development, robust immunogenicity and flexible antigen design over traditional vaccines. This review systematically summarizes the core research progress of mRNA vaccines, including their structural composition with five functional elements and novel subtypes (linear mRNA, self-amplifying RNA, circular RNA) with unique biological characteristics and application value. It elaborates on the immune activation mechanism of mRNA vaccines, which mimic natural viral infection to trigger both innate and adaptive immunity, and analyzes mainstream delivery systems (lipid nanoparticles, dendritic cells, protamine, exosomes, polymers) with their respective performance, advantages and bottlenecks. This review also details the clinical application status of mRNA vaccines in infectious diseases (influenza, rabies, monkeypox, SARS-CoV-2, HIV, parasites) and cancer therapy, highlighting promising preclinical and clinical results of candidate vaccines and combined therapeutic regimens. Additionally, it addresses the current limitations of mRNA vaccines, such as delivery inefficiency, production costs, and cold chain constraints. Finally, this review prospects the future development direction, emphasizing that the optimization of delivery systems, antigen design and production processes will further promote the clinical translation and diversified application of mRNA vaccines in disease prevention and treatment.

## 1. Introduction

mRNA vaccines are a significant milestone in vaccine development. The highly effective protective efficacy of the mRNA vaccines used to combat the pandemic is the result of six decades of technological development. This technological advancement not only expands the application boundaries of mRNA technology, but also lays a core technical foundation for the research and development of mRNA vaccines. mRNA vaccines have demonstrated significant advantages when compared with traditional vaccine technologies such as live vaccines, inactivated vaccines and subunit vaccines. Specifically, mRNA vaccines do not require the cultivation of live pathogens, which not only ensures high safety but also avoids the risk of virulence reversion of attenuated vaccines; they have a short design and production cycle and can quickly respond to emerging infectious diseases. These inherent advantages endow mRNA vaccines with great application potential in multiple disease prevention [1].

In 2020, the successful launch of the COVID-19 mRNA vaccine achieved a revolutionary leap of mRNA therapy from basic research to clinical application, providing a new technical approach for global infectious disease prevention and control. In addition to the widely used COVID-19 mRNA vaccine, the research and development of mRNA vaccines targeted various pathogens such as respiratory viruses, herpes viruses, and influenza [2]. At present, the focus of relevant research is gradually shifting from emergency prevention and control to routine prevention and control, and the broad-spectrum activity and long-term efficacy of vaccines have become key research directions in this field.

Researchers have been dedicated to optimizing the delivery systems and sequence design of mRNA vaccines. Existing studies have confirmed that the length of the coding sequence is recommended not to exceed 4–5 kb, and longer sequences tend to reduce stability [3]. The duration of the antigen expression is also regulated by various factors such as delivery carriers. Conventional LNP-delivered vaccines can maintain antigen expression for 2–4 weeks. This period can be extended to 6–8 weeks through strategies such as sequence modification and carrier optimization, which strongly supports the long-term protective efficacy of vaccines and promotes the rapid development of mRNA vaccine technology [4]. With the continuous iteration and development of mRNA technology, in addition to traditional linear mRNA, a variety of new mRNA molecules have been successfully developed. Circular RNA (circRNA) and self-amplifying RNA (saRNA) belong to the same category of novel RNA molecules with important innovative value.

In recent years, with the rapid development of novel RNA formats such as self-amplifying RNA and circular RNA, the continuous optimization of delivery systems including lipid nanoparticles, dendritic cell-based delivery, and polymeric carriers, as well as the steady progress of clinical trials of mRNA tumor vaccines, relevant cutting-edge technologies and clinical outcomes have formed a new research system. This review systematically summarizes the recent technological breakthroughs and clinical applications of mRNA vaccines, focusing on their design strategies, immune mechanisms, key delivery technologies, and application status in disease prevention and tumor therapy, so as to provide a reference for the further development and practical application of mRNA vaccine-related research.

## 2. Materials and Methods

### 2.1. Search Strategy and Data Sources

Three internationally recognized core databases, including PubMed, Web of Science, and Embase, were selected to comprehensively obtain original research, clinical trial reports, and review articles related to mRNA vaccines worldwide. All database searches were performed through their official standard search interfaces to ensure the standardization of the retrieval process.

### 2.2. Search Terms and Keywords

A combined retrieval mode of “subject terms + free words” was adopted. All retrieval terms were standardized and strictly matched with the thesaurus of each database. The retrieval formula was: (“mRNA vaccine” OR “messenger RNA vaccine” OR “mRNA-based vaccine”) AND (“clinical trial” OR “preclinical study” OR “antigen design” OR “delivery system” OR “immunogenicity” OR “safety” OR “protective efficacy”).

During the retrieval process, the above retrieval formula was first used for cross-matching retrieval to complete the initial literature collection. Subsequently, the reference lists of all included literature were manually traced, and supplementary retrieval was conducted for relevant literature that was not retrieved, so as to further improve the comprehensiveness of literature retrieval and ensure that no core research was omitted.

### 2.3. Eligibility Criteria and Study Selection

Inclusion Criteria: To strictly define the scope of included studies and ensure that the included studies were highly relevant to the theme of this review with complete and reliable data, the following inclusion criteria were formulated: ① study type: peer-reviewed original research papers, clinical trial reports, experimental studies, and review articles directly related to mRNA vaccines, excluding conference abstracts, correspondence letters, and dissertations without complete research data; ② research content: studies focusing on the design, preparation process, antigen screening and optimization, delivery system development and improvement, preclinical evaluation (including immunogenicity, safety, and protective efficacy), clinical trials of various phases, and related mechanisms of action of mRNA vaccines; and ③ data integrity: studies with clear description of research methods, scientific and reasonable experimental design, clear and traceable research results, and complete access to key data (such as neutralizing antibody titer, immune protection rate, and adverse reaction rate) without missing core data.

Exclusion Criteria: To exclude irrelevant and low-quality studies and avoid affecting the scientificity and rigor of this review, the following exclusion criteria were formulated: ① irrelevant research theme: studies that did not involve content related to mRNA vaccines; ② non-standard data: studies with incomplete data, ambiguous research conclusions, obvious defects in experimental design (such as too small sample size, no control experiment, poor experimental repeatability), or unreliable experimental results; and ③ a lack of immune evaluation: animal experiments and in vitro experiments that did not involve the evaluation of immunogenicity, safety, or protective efficacy of mRNA vaccines.

## 3. Design and Structure of mRNA Vaccines

### 3.1. Basic Structure of mRNA Vaccine

**Figure 1:**
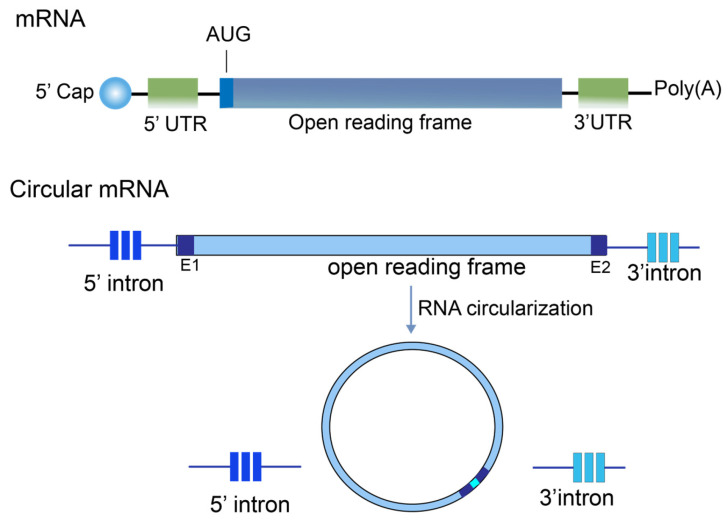
The structure of mRNA and circular RNA.

mRNA vaccines are composed of five typical functional elements: the 5′ cap, the 3′ poly(A) tail, the 5′ and 3′ untranslated regions (UTRs), and the open reading frame (ORF). These elements are arranged in a linear fashion and collectively ensure the key functions of mRNA, including translation efficiency, stability, and targeting (Figure 1).

The open reading frame (ORF) region serves as the coding region of mRNA, and its translational efficiency is of critical importance. Optimizing and modifying the ORF have been a key focus of research in the field of mRNA vaccine development. In terms of optimization strategies, traditional approaches involve replacing rare codons with synonymous high-frequency codons or codons with higher host tRNA abundance. This approach aligns with the codon usage preferences of host genes expressed at high levels while ensuring adequate tRNA supply during the expression of exogenous mRNA, thereby effectively enhancing translation efficiency [5]. The 5′ cap structure of mRNA is located at the 5′ end of mature mRNA in eukaryotic cells. It is connected to the 5′ nucleotide of mRNA via a 5′-5′ triphosphate bridge, forming the m7G cap structure (m7GpppNp). This structure prevents degradation by exonucleases, thereby maintaining mRNA stability and initiating the translation process [6]. The poly(A) tail, which consists of 10 to 250 adenine ribonucleotides, acts as a dynamic addition to mRNA. Its length is crucial for regulating mRNA translation and protein expression. The optimal length of the poly(A) tail can enhance both translation efficiency and mRNA stability. Studies have shown that increasing the length to 120 bp leads to correspondingly higher protein expression levels [7]. Researchers have also developed a method for synthesizing poly(A) tails of a specific length. This enables the precise regulation of tail length and provides a more accurate technical means of optimizing poly(A) tails [8].

### 3.2. New mRNA Subtype

With the continuous iteration and development of mRNA technology, in addition to traditional linear mRNA, a variety of new mRNA molecules have been successfully developed. Self-amplifying RNA (saRNA) and circular RNA (circRNA) belong to the same category of novel RNA molecules with important innovative value. They complement each other synergistically, further enriching the research and development technology system of new mRNA vaccines, laying an important foundation and providing a new technical direction for the diversified development of mRNA vaccines. The differences in three types of RNA molecules are shown in Table 1.

saRNA, which enables self-replication and efficient antigen expression, is a major focus in mRNA vaccine research. Its core feature is containing both an ORF encoding the target antigen and a replication element encoding RNA-dependent RNA polymerase (RdRp), enabling independent self-replication and amplification in host cells without relying on the host’s replication mechanism [9]. Its mechanism is: after entering host cells via delivery systems, saRNA first translates to produce RdRp, which then initiates self-replication to generate numerous progeny saRNAs; each progeny serves as a template for efficient antigen synthesis, achieving sustained antigen expression to stimulate immune responses [10]. Compared with traditional linear mRNA, saRNA has unique advantages: it achieves high-level, long-term antigen expression at extremely low doses, reducing vaccine dosage, production costs, and improving accessibility; its sustained antigen expression stimulates strong, long-lasting humoral and cellular immunity, enhancing vaccine protective efficacy [11]. Currently, saRNA vaccines have achieved phased progress, with extensive basic and clinical studies in infectious diseases (influenza, Zika virus, SARS-CoV-2) and tumor immunotherapy [12,13]. For example, optimized SARS-CoV-2 saRNA vaccines showed stronger immunogenicity and longer immune protection than traditional mRNA vaccines in animal experiments [14]. Tumor-targeted saRNA vaccines can activate anti-tumor immunity, inhibiting tumor growth and metastasis [15]. Despite existing challenges (e.g., insufficient intracellular delivery efficiency, difficult precise regulation of immunogenicity), saRNA remains a key direction for future mRNA vaccine development with broad clinical prospects.

Circular RNA (circRNA) is a novel class of RNA molecules whose formation mechanism differs significantly from that of traditional linear RNA. It is primarily formed through a reverse splicing process involving exons or introns of precursor mRNA. During the biosynthetic stage, the 3′ end of the downstream exon attacks the 5′ end of the upstream exon, thereby forming a circular structure with a 5′-3′ covalent linker tail [16]. This unique formation method gives circRNA a stable molecular structure and lays the molecular foundation for its diverse coding and non-coding functions within cells (Figure 1). Due to its unique structure, circRNA plays a significant regulatory role in non-coding functional domains, most notably by acting as a microRNA (miRNA) sponge to regulate gene expression. A classic example is ciRS-7 (the antisense strand of the gene for the protein cerebellar degeneration-related protein 1, CDR1as). This circRNA contains over 70 conserved binding sites for miR-7 [17]. By binding specifically to miR-7, ciRS-7 inhibits its activity, thereby lifting the suppression of target protein expression by miR-7 and ultimately achieving the regulation of disease progression [18,19]. In addition to their non-coding functions, researchers have increasingly elucidated the coding potential of circRNAs. CircRNAs were first confirmed to encode and translate proteins in fruit flies in 2015 [20], laying critical theoretical foundation for circRNA vaccine development. Unlike linear mRNA, circRNA lacks 5′ cap and 3′ tail structures, so its translation initiation is independent of the 5′ cap, but occurs in vivo and in vitro via N6-methyladenosine (m6A) modification or internal ribosome entry sites (IRESs) [21,22].

In summary, research on the translation mechanisms and functional characteristics of linear mRNA, circRNA, and saRNA has laid a solid foundation for in vivo applications of engineered novel RNA molecules and provided new ideas and technical support for the diversified development of mRNA vaccines.

## 4. Mechanism of mRNA Vaccines

**Figure 2:**
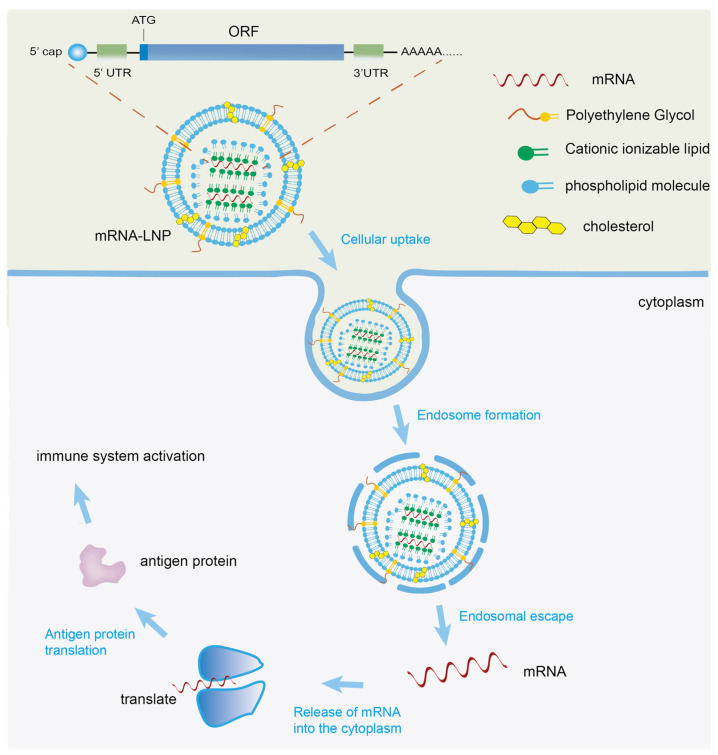
Construction and mechanism of mRNA vaccine.

mRNA vaccines initiate immune activation through multiple sequential steps to complete the immune response chain. mRNA enters the body via different routes, such as an intramuscular or subcutaneous injection. These mRNA are then actively captured by antigen-presenting cells or absorbed by somatic cells at the injection site and subsequently transported into the cytoplasm via intracellular transport mechanisms. Once in the cytoplasm, the mRNA undergoes translation via ribosomes to produce the corresponding antigen proteins. These proteins then undergo post-translational modifications and enter secretory pathways, cell membranes or other subcellular compartments via target sequences or transmembrane domains to form functionally active mature proteins. Some of these proteins are released into the extracellular space, while others are processed by lysosomes into small antigenic peptides within the cell [23,24] (Figure 2).

These processes closely mimic the natural infection pathway of RNA viruses in the body, first activating the innate immune response and then gradually guiding the initiation of the adaptive immune response [25]. Antigen presentation, as the key link between innate and adaptive immunity, plays a central role in this process. Antigen peptides generated through lysosomal processing are presented to CD8^+^ T cells via the MHC class I pathway and to CD4^+^ T cells via the MHC class II pathway. Meanwhile, intact antigen proteins released into the extracellular space can be directly recognized by B cells. This multi-pathway antigen recognition model enables the simultaneous activation of cellular immunity and humoral immunity [26], achieving the comprehensive initiation of the body’s immune response. The advantages of mRNA vaccines in antigen expression kinetics also distinguish them significantly from protein vaccines, subunit vaccines, and viral vector vaccines. Specifically, mRNA vaccines can reach the peak of antigen expression within 24~48 h after injection and maintain an effective antigen level for 7~14 days, while protein vaccines and subunit vaccines usually require 3~7 days to reach the peak of antigen expression with an effective duration of only 3~5 days; viral vector vaccines have similar antigen expression kinetics to mRNA vaccines, but their peak expression level is only 60%~80% of that of mRNA vaccines, and they are limited by pre-existing antibodies in the body, which affects the immune effect [27].

Specifically, the immune sensing system plays a role in regulation by recognizing the characteristics of mRNA molecules. Unmodified single-stranded RNA and its degradation products are recognized by Toll-like receptor 7 (TLR7) [28]. Meanwhile, mRNA modified with nucleosides, such as the introduction of pseudouridine and its m1Ψ derivatives, enhances translation efficiency and increases stability and half-life [29]. It also significantly reduces the activation of innate immune sensors, such as RIG-1 and melanoma-associated protein 5 (MDA5) [30]. This difference induced by modification is evident in actual research, such as influenza virus vaccine experiments, where mRNA-iLNP-encoded vaccines only elicit strong T cell and germinal center immune responses when the mRNA is modified with m1Ψ. Unmodified mRNA fails to achieve this effect, directly demonstrating that the molecular characteristics of mRNA influence the intensity of immune activation through the immune sensing system [31]. In terms of immunological adjuvant synergy, the nano-adjuvant system developed by the Chinese Academy of Sciences in 2025 is a prime example. This system uses mesoporous silica nanoparticles modified with cationic polymers to deliver mRNA and TLR7 agonists together. In mouse models, the system significantly enhances the uptake and presentation of antigens by dendritic cells, leading to the activation and proliferation of CD8^+^ and CD4^+^ T cells. Th1-type cytokine secretion increases three- to five-fold, effectively activating both cellular and humoral immune responses [32]. Compared with other mainstream adjuvant strategies, this system has better biocompatibility and antigen delivery efficiency than traditional aluminum adjuvants, and its ability to enhance cellular immunity is superior to that of the lipid nanoparticle adjuvant system (LNP), but its preparation process is more complex than that of LNP, and the cost is 2~3 times higher; in addition, although this system has achieved good results in mouse models, its repeatability in large animal models has not been verified, and the potential clinical transformation obstacles include high difficulty in large-scale production, possible long-term biosafety risks, and unclear regulatory approval standards, all of which need to be further addressed in subsequent studies.

Meanwhile, significant progress has been made in clinical combination therapy studies. A Phase I clinical trial conducted by the American Cancer Centre in 2024 demonstrated that, in the treatment of pancreatic ductal adenocarcinoma, combining the mRNA neoantigen vaccine *autogene cevumeran* with the PD-L1 inhibitor Atezolizumab was more effective than using either treatment alone. Fifty percent of patients exhibited a strong neoantigen-specific CD8^+^ T cell immune response, and there was an 86 percent reduction in the risk of disease recurrence compared to non-responders. This confirms that combination therapy can reshape the tumor microenvironment and enhance T cell killing activity [33]. However, it has a small sample size, lacks a randomized controlled design, and has immature survival data, so the long-term efficacy and safety still need to be verified by larger-scale subsequent trials.

Overall, the research progress of mRNA vaccines in sequence optimization, adjuvant synergy, and clinical combination therapy has gradually deepened people’s understanding of their immune activation mechanisms and significantly improved the overall efficacy of vaccines. However, both the limitations of clinical combination therapy and the technical development itself determine that its progress is not isolated, but faces fierce competition from other vaccine technology platforms. Among them, viral vector vaccines (such as adenoviral vectors and lentiviral vectors) have mature clinical transformation experience and are widely used in gene therapy-related vaccine fields, but they are limited by pre-existing antibodies in the body and the risk of insertional mutation, resulting in limited application scenarios; recombinant protein vaccines have high safety and strong clinical recognition, but their production cycle is long (4~6 months) and production cost is relatively high, making it difficult to quickly respond to the prevention and control needs of sudden infectious diseases; DNA vaccines have a simple preparation process and low cost, and are easy for large-scale production, but their transfection efficiency is low and immune effect is weak, requiring the further optimization of delivery systems; protein nanoparticle vaccines have good targeting and biocompatibility, but their antigen loading capacity is lower than that of mRNA vaccines, making it difficult to meet the needs of high-antigen demand scenarios [34,35].

In the future, leveraging its advantages of fast research and development speed, high immune efficiency, and rapid iteration, mRNA vaccines will show irreplaceable advantages in acute infectious disease prevention and control and personalized tumor vaccines; in contrast, recombinant protein vaccines, viral vector vaccines, and other vaccine platforms will still play an important role in chronic disease prevention and gene therapy, ultimately forming a diversified and differentiated vaccine development pattern.

## 5. mRNA Vaccine Delivery System

As a negatively charged macromolecule, mRNA has difficulty penetrating anionic cell membranes [36], so a delivery system is needed to protect mRNA, promote cellular uptake, and achieve endosomal escape in order to achieve the desired effect. Currently, researchers have developed delivery systems such as lipid nanoparticles, protamine, engineered exosomes, polymer carriers, and dendritic cells (Table 2).

### 5.1. Lipid Nanoparticle

Lipid nanoparticles (LNPs) are typically composed of ionizable lipids, polyethylene glycol (PEG) lipids, neutral lipids, and cholesterol [37]. PEG localizes on the LNP surface to prevent aggregation and immune cell phagocytosis by inhibiting nonspecific interactions with serum components [38,39]. Cholesterol enhances membrane fusion to facilitate mRNA entry into the cytoplasm, while neutral auxiliary lipids (mainly saturated phospholipids) increase the phase-transition temperature and stabilize lipid bilayers [40,41]. Ionizable cationic lipids represent the most critical excipients, as they directly determine mRNA delivery and transfection efficiency [42]. LNPs have greatly promoted the development of COVID-19 mRNA vaccines, typified by the Pfizer-BioNTech Comirnaty and Moderna Spikevax, both of which employ optimized four-component LNP systems [43]. Despite their advantages of low immunogenicity, high cargo capacity, and scalable production, LNPs still face critical bottlenecks restricting clinical translation, including batch-to-batch heterogeneity, complement activation-related pseudoallergy (CARPA), anti-PEG antibody induction, and low endosomal escape efficiency, all of which impair delivery performance and vaccine consistency [44,45].

To overcome these limitations, continuous innovation in LNP design has been achieved. In 2024, Lu et al. developed charge-assisted stability (CAS) technology to construct negatively charged CAS-LNPs, which resist aggregation and fragmentation during nebulization, thus enabling efficient lung delivery with approximately 20-fold higher mRNA expression than clinical formulations [46]. Also, in 2024, Wei et al. optimized a three-component stLNP system with enhanced delivery efficiency and organ specificity, and achieved controlled expression in target cells by incorporating miRNA-responsive sequences, showing precise therapeutic potential in melanoma lung metastasis [47]. In 2025, Yang et al. designed disulfide-containing SLNPs (S-DOPE) that utilize thiol-mediated dynamic covalent exchange to bypass the endosomal entrapment bottleneck, yielding 11-fold higher in vitro translation efficiency and 4.5-fold enhanced in vivo mRNA expression and immune responses [48]. With deeper mechanistic insights and large-scale clinical validation, LNPs will continue to serve as a core delivery platform for next-generation mRNA vaccines and therapeutics.

### 5.2. Dendritic Cell-Loaded Delivery System

Dendritic cells (DCs) are ideal mRNA vaccine targets, as these professional antigen-presenting cells can internalize, process, and present antigens to immune cells to induce effective adaptive immunity [49], while also secreting chemokines to recruit T cells [50], a capacity confirmed since the 1990s, when studies demonstrated DCs could reliably activate in situ T cells that recognize MHC molecules on initially activated DCs [51]. The main mRNA loading methods for DCs are ex vivo (isolating immature DCs from peripheral blood, maturing and loading them with antigen-encoding mRNA, then reinfusing) and in situ (directly injecting antigen-encoding mRNA complexed with TriMix into lymph nodes), with clinical trials (NCT01684241) showing that TriMix outperforms other stimulatory cytokines in activating DCs and enhancing effector T cell function in advanced melanoma patients [52]. Recent technological breakthroughs include Cai et al.’s 2024 design of MS2 stem-loop-structured mRNA modified with Sindbis virus glycoprotein to target DC-SIGN, significantly improving antigen mRNA delivery efficiency, DC lymph node migration, and cellular immunity activation [53], and Cheng et al.’s albumin-based EB-LNP with high lymphatic drainage to target lymph nodes and reduce liver accumulation [54]. Despite challenges such as high ex vivo DC preparation costs and insufficient in situ targeting precision, continuous innovation in mRNA loading and DC-targeting technologies promises the clinical translation of DC-based mRNA vaccines.

### 5.3. Protamine Delivery System

Protamine, a natural cationic polypeptide mixture, is a promising mRNA delivery vehicle with two key advantages: its high positive charge enables spontaneous complexation with negatively charged mRNA to protect it from serum nuclease degradation [55], and it acts as an adjuvant—protamine–mRNA complexes are recognized by immune cells via the TLR-7/TLR-8 pathway, activating robust immune responses and the secretion of cytokines like type I interferon, TNF-α, and IL-12 [56,57]. Protamine–mRNA nanoparticle size (regulated by protamine–mRNA ratio and diluent salt concentration [58]) modulates immune stimulation [59,60], with particles <450 nm effectively activating pDCs to release IFN-α [61,62]. Widely used in clinical trials for rabies and non-small-cell lung cancer [63], protamine has lower transfection efficiency than LNPs due to poor cell membrane penetration and endosomal escape [55,64,65], a bottleneck addressed by liposomal encapsulation to enhance formulation stability and immunogenicity [66]. With further mechanistic research and clinical validation, protamine will remain a safe, adjuvant-equipped mRNA delivery platform.

### 5.4. Engineering Exosomes

Exosomes, naturally secreted by various cells (e.g., immune, cancer, stem cells) [67], are promising mRNA delivery vehicles due to their ability to encapsulate mRNA. Their lipid bilayer protects encapsulated mRNA from extracellular enzyme degradation [68], while the biological membrane enables evasion of macrophage degradation to extend in vivo circulation [69]. Exosomes can cross biological barriers, promote their own uptake by recipient cells, enhance cargo loading efficiency [70], and release cargo directly into the cytoplasm via membrane fusion, with superior biocompatibility and fewer adverse effects compared to synthetic carriers [71]. Endowed with donor cell surface proteins for tissue-specific targeting [72], their targeting can be further enhanced via genetic, chemical, or enzymatic modification [73,74]. Recent advances include Zhuo et al.’s cholesterol-enriched milk exosomes improving delivery and anti-tumor efficacy [75], and Du et al.’s dual-modified exosomes achieving safe, effective Wnt2 mRNA delivery [76]. Despite challenges in isolation standardization and batch consistency, optimized large-scale production and engineering will promote their clinical translation.

### 5.5. Polymer Carrier Delivery System

Polymers are long-chain macromolecules composed of hundreds to tens of thousands of repeating units. Endowed with a positive charge, they can form stable complexes with negatively charged mRNA through strong interactions, which are phagocytosed by cells to release mRNA after endosomal escape for translation [77]. As mRNA delivery vehicles, they must be non-toxic, biodegradable, and biocompatible, with types expanding from early cationic polymers (e.g., polylysine) to PEI, PAMAM and PLL [78,79]. Compared to liposomes, polymers have higher transfection and RNA loading efficiency [80], as demonstrated by Tan et al. who delivered DUSP5 siRNA to cardiac tissue via linear PEI to improve cardiomyopathy [81]. They also enhance RNA stability and are non-immunogenic, though challenges like insufficient endosomal escape and potential biodegradation residues exist. Molecular modification and composite strategies will help overcome these bottlenecks for broader clinical application.

## 6. mRNA Vaccines for Cancer and Infectious Disease

### 6.1. Cancer mRNA Vaccine

To date, more than 120 clinical trials of mRNA tumor vaccines have been conducted worldwide, of which over 80 focus on cancer mRNA vaccines, covering lung cancer, colorectal cancer, melanoma, pancreatic cancer, breast cancer, cervical cancer and other major tumor types, fully reflecting the broad research scope and active development momentum in the field of cancer vaccines.

In the field of tumor therapy, a Phase I clinical trial of the mRNA neoantigen vaccine autogene cevumeran (BNT122) showed that it can continuously reduce the risk of disease recurrence in patients with surgically resected pancreatic ductal adenocarcinoma (PDAC), and the recurrence risk of patients responding to the vaccine is 86% lower than that of non-responding patients [82]. In addition, a Phase I clinical trial of the mRNA vaccine targeting HER2 (mRNA-HER2) for the treatment of advanced breast cancer showed that 7 out of 22 enrolled patients achieved stable disease, with a disease control rate (DCR) of 31.8%, providing a new therapeutic option for patients with HER2-positive breast cancer who are resistant to traditional therapy [83]. In the field of infectious diseases related to cancer, mRNA vaccines targeting human papillomavirus (HPV) types 16 and 18, which are closely associated with cervical cancer, have entered Phase III clinical trials, and their efficacy in preventing HPV infection and reducing the incidence of cervical intraepithelial neoplasia has been fully verified [84]. A Phase I/II clinical trial of personalized mRNA neoantigen vaccines combined with chemotherapy for non-small-cell lung cancer (NSCLC) showed that the overall response rate (ORR) of the combined group reached 58.3%, and the median overall survival (OS) was prolonged by 6.5 months compared with the chemotherapy-alone group [85]. Additionally, an early assessment showed that a lipid-based vaccine combined with CAR-T therapy for recurrent, refractory advanced solid tumors (targeting CLDN6) resulted in partial remission in 57% of patients [86]. Furthermore, animal studies have confirmed that CXCR4-targeted p53 mRNA nanoparticles combined with anti-PD-1 therapy are more effective [87]. These clinical data indicate that mRNA tumor vaccines have demonstrated certain therapeutic potential, and some combined treatment regimens have achieved phased results in small-sample clinical trials, serving as an important support for their advancement towards clinical application.

In terms of DC vaccines, research has shown that co-transfection of mRNA encoding immunomodulatory factors (such as TriMix) via electroporation can enhance immune responses [88,89]. For instance, combining TriMix DC vaccines with ipilimumab to treat advanced melanoma has successfully induced tumor-associated antigen-specific CD8^+^ T cell responses [90]. In the field of dendritic cell (DC) vaccine optimization, Fu et al. developed a new generation of mRNA lipid nanoparticle (LNP) vaccines that encode membrane-type IL-12 (mtIL-12) as an adjuvant. In various tumor models, this vaccine effectively avoids the systemic side effects associated with traditional IL-12 while inducing potent tumor-specific T cell responses. This leads to the formation of a population of pre-effector T cells with enhanced tumor-killing capacity in draining lymph nodes. This provides a novel strategy for optimizing DC vaccines and enhancing the efficacy of mRNA tumor vaccines overall [91].

Currently, although mRNA tumor vaccines have initially been feasible for clinical application, three core bottlenecks still need to be broken through: first, expanding the sample size of clinical trials to verify the stability and safety of vaccine efficacy and solve the problem of efficacy fluctuations in small-sample trials; second, optimizing delivery systems and vaccine formulations to further improve vaccine delivery efficiency, enhance tumor-specific immune responses, and reduce systemic side effects; and third, achieving the large-scale production of personalized vaccines to reduce research and development and treatment costs and improve clinical accessibility. As our understanding of tumor-specific antigens deepens, cancer vaccine research is becoming an increasingly popular topic in the field, driving ongoing breakthroughs in personalized therapy and technological optimization [92,93]. It is expected that some mature mRNA tumor vaccines will be approved for clinical application in the next 3–5 years, gradually achieving the transition from “clinical trials” to “clinical popularization.”

### 6.2. Virus mRNA Vaccine

#### 6.2.1. Influenza mRNA Vaccine

Several influenza mRNA vaccines are currently in the clinical development stage. Moderna’s mRNA-1851 (targeting H7N9) and mRNA-1440 (targeting H10N8) have shown promising results in Phase I trials, with the former achieving seroconversion rates of 96.3% (HAI) and 100% (MN) at a dose of 50 μg for H7N9 and the latter achieving seroconversion rates of 78.3% (HAI) and 87%. Moderna’s quadrivalent mRNA-1010 vaccine (containing H1N1, H3N2 and two B-type strains) has entered Phase III trials with over 30,000 participants [94]. The vaccine demonstrated 67.2% protective efficacy against seasonal influenza in the 18–64 age group, which is an improvement of 18.5% over traditional vaccines. Specifically, it demonstrated 72.3% protective efficacy against the H3N2 variant [95]. Pfizer’s quadrivalent mRNA vaccine completed Phase III trials in individuals aged 65 and over, demonstrating 73.1% efficacy in preventing severe influenza, and no serious adverse reactions were reported [96]. In 2024, SinoVac’s trivalent influenza mRNA vaccine (H1N1, H3N2, Victoria lineage) entered Phase II clinical trials in China, using a self-developed ionizable lipid carrier. Preclinical data showed a protection rate of 100% against homologous strains and 65% against heterologous strains [97].

However, the clinical development and practical application of influenza mRNA vaccines still face three key issues that have not been fully analyzed: first, the difference in real-world effectiveness between different seasons. Due to differences in temperature, humidity, and population mobility across seasons, the transmission intensity of influenza viruses varies significantly, leading to obvious differences in the real-world protective efficacy of influenza mRNA vaccines. For example, the protective efficacy in winter and spring (high-incidence seasons) is generally 10–15% higher than that in summer and autumn (low-incidence seasons), which is mainly related to the higher viral exposure dose in high-incidence seasons [98]. Second, antigenic drift is a major challenge affecting the long-term effectiveness of influenza mRNA vaccines. Influenza viruses are prone to subtle genetic mutations during replication, resulting in antigenic drift, which makes the antigens encoded by the vaccine mismatched with the circulating strains in the population, thereby reducing the protective effect of the vaccine [99]. To address this issue, the World Health Organization (WHO) recommends updating influenza vaccine strains twice a year based on global influenza surveillance data to improve the matching degree between vaccine strains and circulating strains. Third, the regulatory evaluation criteria for influenza mRNA vaccines, especially the non-inferiority/superiority comparison with traditional inactivated influenza vaccines, have not been clearly clarified in current research. Nevertheless, the rapid development and iterative advantages of mRNA technology provide feasible solutions to these problems, and with the continuous accumulation of clinical data and improvement in regulatory standards, influenza mRNA vaccines are expected to play a more important role in global influenza prevention and control.

#### 6.2.2. Rabies Virus mRNA Vaccine

Recent years have witnessed continuous breakthroughs in rabies mRNA vaccine development. Preclinically, Li et al. showed that LVRNA001 provided safe, effective protection for dogs, mice and crab-eating macaques [100], while a study found RABV-Full and RABV-R333Q vaccines induced complete protection, and heterologous immunization combined IRV and mRNA vaccine advantages [101]. Clinical trials include protamine-conjugated CV7201 (safe but with administration-dependent immune responses [63,102,103]) and LNP-formulated CV7202 (more effective, with low doses inducing WHO-standard antibodies). Despite these encouraging findings, challenges still exist in delivery efficiency, long-term immune persistence, and administration standardization. Further optimization of antigen design, delivery systems, and immunization protocols is warranted to achieve effective global rabies control [104].

#### 6.2.3. Monkeypox Virus mRNA Vaccine

The monkeypox virus (MPXV), a double-stranded DNA virus of the Orthopoxvirus genus in the Poxviridae family, exists as intracellular mature viruses (IMVs, carrying M1R, E8L, A29L, H3L [105,106]) and extracellular envelope viruses (EEVs, IMVs with endoplasmic reticulum-derived envelopes and A35R, B6R [107]), providing key targets for vaccines. Following the principle that combined IMV and EEV antigens enhance protection [108], monkeypox mRNA vaccines adopt multi-antigen strategies: China’s 2022 VGPox1-3 (targeting M1R and A35R) showed fusion vaccines outperformed the mixed one [109]. Clinical translation has advanced rapidly, with BioNTech’s 2025 Phase I data (BNT166a/c, NCT05988203) showing high safety and 100% neutralizing antibody induction, and BNT166c (with B6R) inducing 35% stronger cellular immunity [110]. Optimized antigen design, delivery and immunization protocols are expected to make them reliable for global monkeypox control.

#### 6.2.4. Zika Virus mRNA Vaccine

Zika virus, a neurotropic virus first identified in Uganda’s Zika Forest in 1947 and transmitted via mosquito bites and sexual contact, causes severe complications including placental dysfunction, congenital malformations and Guillain–Barré syndrome, with no approved vaccine currently [111]. Several Zika mRNA vaccines (e.g., mRNA-1893, mRNA-1325, LNP-formulated one in NCT03014089) are in development [112]. Richner et al. found prM-E-encoding modified mRNA-LNP induced high neutralizing antibodies, and E protein mutations addressed cross-reactivity risks and prevented vertical transmission [113]. saRNA vaccines, effective at low doses, and 2024 circRNA vaccines targeting EDIII also show promise, indicating mRNA-related technologies offer safe, immunogenic strategies for Zika vaccine development [114,115].

#### 6.2.5. SARS-CoV-2 mRNA Vaccine

Continuous innovation in mRNA delivery systems (e.g., LNP technology) and nucleotide modification has achieved remarkable breakthroughs in SARS-CoV-2 infection prevention, and the advantages of rapid development and efficient production of this technology have enabled multiple mRNA vaccines to quickly enter clinical application or trial phases, providing critical technical support for the global fight against the pandemic. Representative vaccines include Pfizer-BioNTech’s BNT162b2, the first US-approved mRNA vaccine encoding a full-length spike glycoprotein with two proline mutations to enhance immunogenicity [116]; its Phase III trial involving 43,548 participants showed 95% overall efficacy after two doses, with variant-specific efficacies of 89.5% (Alpha), 75.0% (Beta) and 51.9% (Delta), and a three-dose regimen can enhance cross-protection against Delta and Omicron variants [117]. Moderna’s mRNA-1273, which encodes the full-length SARS-CoV-2 spike fusion protein, exhibits high efficacy against Alpha (100%) and Beta (96.4%) variants [118,119], with efficacy against Delta increasing to 95.2% and against Omicron decreasing to 62.5% after three doses, while two doses provide 94% efficacy against severe COVID-19 [120,121]. Additionally, PTX-COVID19-B and ARCoV have shown good immunogenicity by inducing high-titer neutralizing antibodies [122,123], and all these vaccines have controllable safety with mostly mild-to-moderate adverse events and low allergic reaction rates, while variant-specific formulations and bivalent strategies are being explored to address viral immune escape [124].

#### 6.2.6. HIV mRNA Vaccine

Since its discovery in 1981, AIDS, a fatal chronic disease caused by HIV infection, has lacked an effective and economical cure, but the emergence of mRNA vaccine technology has brought new solutions to this global public health challenge. Currently, several HIV mRNA vaccines are in clinical research: although the ex vivo loading-based direct delivery system could safely induce antigen-specific CD4^+^ and CD8^+^ T cell immune responses in early stages, a previous study by Gandhi et al. showed that autologous dendritic cell vaccines transfected with HIV-1 Gag and Nef mRNA only elicited transient and weak immune responses, highlighting the urgent need for delivery system innovation [125]. Benefiting from the success of SARS-CoV-2 mRNA vaccines, HIV mRNA vaccine development has accelerated significantly in recent years, with ongoing clinical trials including mRNA-1574, HVTN 302 (NCT05217641) investigating three candidates based on BG505 MD39 trimer stable spike protein [126,127], and IAVI G003 (NCT05414786) and IAVI G002 (NCT05001373) evaluating strain-targeting eOD-GT8 60 mer (mRNA-1644) and Core-G28V2 60 mer [128]. In 2025, William Schief’s team found that membrane-bound HIV mRNA vaccines induced relevant antibodies in ~80% of participants, compared to only 4% for non-membrane-bound ones, as they mimic natural infection, resemble viral structure, expose key antigenic sites, and induce more neutralizing antibodies targeting vulnerable sites on HIV envelope trimer [129]. With continuous innovation in antigen design and delivery systems, mRNA vaccines remain one of the most promising strategies for developing a safe and effective HIV vaccine.

### 6.3. Parasite mRNA Vaccine

In the field of vaccine research targeting specific parasite, progress has been made in the study of *Toxoplasma gondii*. Chahal et al. [130] developed a hexavalent mRNA vaccine using mRNA replicon technology encapsulated in dendritic nanoparticles. The vaccine targets conserved proteins expressed across multiple life cycle stages of *Toxoplasma gondii*, including GRA6, ROP2A, ROP18, SAG1, SAG2A, and AMA1. Animal experiments showed that a single 40 μg vaccine dose enabled test animals to survive for over six months after infection with a lethal dose of *Toxoplasma gondii*. Wu et al. innovatively developed a self-replicating mRNA vaccine targeting *T. gondii* multi-stage-specific antigens (ROP18, TGME49_237490, TGME49_268230, and MIC13) using an LNP-mRNA delivery system. Animal experiments demonstrated that the vaccine induces efficient humoral and cellular immune responses in mice. The vaccine significantly reduces the parasite load in mice, with protection rates against the RH virulent strain, ME49, and WH6 attenuated strains reaching 60%, 80%, and 60%, respectively. Against PRU oocyst challenge, the oocyst reduction rate reaches 72.5%, achieving multi-stage prevention and control against acute infection, chronic cyst formation, and oocyst transmission stages of *Toxoplasma gondii* [131].

Malaria is another severe parasitic disease that causes over 200 million clinical cases and results in hundreds of thousands of deaths each year. Significant progress has also been made in research on mRNA vaccines for malaria. Luo et al. recently developed a novel chemokine fusion mRNA vaccine that targets immature dendritic cells (iDCs). This vaccine significantly enhances the immune response to the cyclin protein of Plasmodium falciparum (PfCSP). The research team constructed mRNA-LNP formulations and evaluated the immune response in mice using animal experiments and an enzyme-linked immunosorbent assay (ELISA). The results showed that mice vaccinated with the chemokine fusion mRNA-LNP formulation had significantly higher titers of anti-NANP6 peptide-specific antibodies than other control groups. Furthermore, this iDC-targeted vaccine was found to effectively enhance resistance to Plasmodium invasion in mice, particularly demonstrating superior efficacy in protecting the liver from infection [132]. Meanwhile, Scaria and his team developed mRNA vaccines that express the malaria transmission-blocking antigens Pfs25 and Pfs230D1. In mouse models, the vaccine induced a strong immune response, with the mRNA construct demonstrating significantly higher functional activity in reducing transmission compared to the corresponding alum-adjuvanted protein conjugate vaccine. Of these vaccines, the Pfs25 mRNA vaccine with a glycosylphosphatidylinositol (GPI) anchor or transmembrane (TM) domain was particularly notable, maintaining over 99% transmission-blocking activity for up to 126 days [133]. Despite these encouraging preclinical advances, the development of mRNA vaccines against parasitic diseases still faces challenges such as complex parasite life cycles, high antigen diversity, and insufficient clinical validation. With continuous optimization of antigen selection and delivery systems, mRNA vaccines are expected to provide new effective weapons for the global prevention and control of parasitic diseases.

In recent years, the development of mRNA vaccines has gained significant momentum, achieving breakthroughs in multiple therapeutic areas. Currently, mRNA vaccines targeting various infectious diseases, including influenza virus, Zika virus, and rabies virus, have entered the clinical trial phase [63,111,112,134,135,136] (Table 3). These candidate vaccines have demonstrated good safety and immunoprotective efficacy in preclinical studies. This provides new technical support and control measures for global public health security.

## 7. The Limitations of mRNA Vaccines

Although mRNA therapy has demonstrated transformative potential in fields such as infectious disease control and cancer treatment, its development still faces multiple critical limitations that need to be overcome. As discussed in previous sections on delivery systems, safety variations, toxicity risks, and the insufficient scalability of different carriers collectively constitute major constraints for the clinical application of mRNA vaccines. Cationic polymer carriers are hampered by potential cytotoxicity and inflammatory responses; exosome-based delivery lacks standardized preparation and quality control protocols; even the widely used lipid nanoparticles (LNPs) still face challenges in formulation stability and local safety. These delivery-related drawbacks, combined with the inherent instability and degradability of mRNA, severely limit the in vivo performance and clinical applicability of the vaccines. In terms of production, although in vitro transcription (IVT) technology has enabled safer and faster manufacturing processes, the cost of core materials remains prohibitively high. The purification process is also highly complex, involving multiple steps such as precipitation, affinity chromatography and ion exchange chromatography to remove impurities such as enzymes, nucleotides and plasmid templates. Stringent purity requirements further increase the difficulty and cost of production. Furthermore, although IVT technology is suitable for small-scale laboratory production, it struggles to meet the yield demands of large-scale mass production, creating a significant bottleneck that hinders its broader application.

Storage and distribution limitations are equally significant. mRNA vaccines have extremely stringent cold chain requirements. Consider the Pfizer/BioNTech SARS-CoV-2 vaccine, for example, it must be transported and stored at temperatures below −70 °C. Even at standard refrigeration temperatures of 2–8 °C, it can only be stored for five days. Establishing a complete cold chain system poses significant technical and financial challenges. Additionally, large-scale global supply relies on sufficient filling capacity, and domestic production capacity alone cannot meet global demand, which directly limits the adoption in less developed regions.

## 8. The Prospects of mRNA Vaccines

mRNA technology, with its highly efficient antigen-updating capability, has emerged as a strategic solution for tackling emerging pathogens. Unlike traditional whole-pathogen or live-virus vaccine technologies, mRNA vaccines utilize non-infectious synthetic genetic templates to express antigens in situ. This characteristic enables multiple technological breakthroughs. Not only does it successfully bypass the inherent limitations of cell membrane translocation, it also supports the precise design of complex multimeric antigens, unrestricted by viral packaging capacity.

In terms of production and application, the fully synthetic process of mRNA vaccines eliminates the need for bioreactors entirely, enabling rapid deployment within days of completing pathogen gene sequencing. The modular platform also has the capability to adapt antigens dynamically, enabling a flexible response to sequence-divergent variants through codon frame adjustments while ensuring consistency across different production batches.

Perhaps most importantly, mRNA vaccines offer significant biosafety advantages. They effectively prevent the production of infectious particles, and, because their transcriptional activity does not integrate into the host genome, they significantly reduce the genetic toxicity risks typically associated with viral vector systems. These technical features make mRNA technology a revolutionary solution for addressing the threat of antigenic variation and enhancing global pandemic preparedness. With the increasing threat of pathogen variation and the rising demand for global pandemic prevention and control, multiple vaccination strategies and combined vaccination modes of various types of vaccines have become important exploration directions in the clinical application of mRNA vaccines. Based on the technical characteristics and application prospects of mRNA vaccines, the advantages and disadvantages of their multiple vaccination strategies and alternative schemes need to be systematically evaluated from multiple dimensions, including immune effect, safety, production application and clinical demand. The core advantages of multiple vaccinations lie in enhancing immune responses, making up for insufficient delivery efficiency, and adapting to the needs of personalized prevention and treatment; however, they also have drawbacks such as cumulative adverse reactions, increased prevention and control costs, enhanced cold chain pressure, and reduced vaccination compliance. To avoid the above problems, alternative schemes such as long-acting mRNA vaccines and circRNA vaccines are currently being explored. As an important improvement in traditional mRNA technology, circular RNA (circRNA) builds on the aforementioned biosafety advantages of traditional mRNA while demonstrating superior technical characteristics. Unlike linear mRNA, circRNA has no free ends at the 5′ and 3′ ends because its circular structure is covalently closed. This enables it to resist degradation by nucleases and significantly prolongs its half-life in the body. This means that lower doses and fewer administrations are required to maintain sustained protein expression and immune responses, providing stronger technical support for improving the efficacy of, and expanding the clinical applications of, RNA vaccines.

Overall, despite remaining challenges in delivery safety, large-scale production, and cold-chain storage, the success of mRNA vaccines in the COVID-19 pandemic has fully validated their core strengths: rapid response to mutations and efficient immune induction. In the future, by optimizing production processes, innovating safe and scalable delivery systems including low-toxicity polymers, standardized exosomes, and next-generation LNPs, and upgrading cold-chain and distribution technologies, mRNA therapy is expected to achieve breakthroughs in precision medicine against a broader range of diseases.

## 9. Conclusions

This article systematically reviews the relevant research achievements, core technologies, and development progress of mRNA vaccines, sorting out the core research hotspots, key technical bottlenecks, and existing controversies of mRNA in terms of molecular mechanisms, delivery systems, and application. Based on existing studies, it can be seen that mRNA technology, with its advantages of strong specificity, short research and development cycle, and wide application range, has formed a relatively clear research framework in fields such as vaccine development and disease treatment, and achieved a series of breakthrough results. However, it also faces practical problems such as insufficient delivery efficiency, difficulty in regulating immunogenicity, and unproven long-term safety. In summary, the field of mRNA vaccine still has broad development space. In the future, focus can be placed on the optimization of delivery systems, precise regulation of immunogenicity, and expansion of multi-field applications to fill the gaps in existing research, promote the advancement of mRNA technology towards more mature and extensive application scenarios, and provide a solid reference for subsequent related basic research and clinical transformation.

## Figures and Tables

**Table 1:** Comparison of practical application characteristics among linear mRNA, circRNA and saRNA vaccines.

	Linear mRNA	Circular RNA (circRNA)	Self-Amplifying RNA (saRNA)
Dose	Relatively high; a sufficient dose is required to achieve effective antigen expression	Moderate; high stability allows effective antigen expression without excessively high doses	Extremely low; the self-replication characteristic enables high-efficiency expression at low doses
Expression Duration	Relatively short; usually short-term expression, requiring multiple administrations to maintain efficacy	Longest; the circular structure resists nuclease degradation, enabling long-acting antigen expression	Relatively long; self-replication can maintain sustained antigen expression and reduce the number of administrations
Preparation Difficulty	Relatively low; simple structure (containing 5′ cap, 3′ poly(A) tail, ORF and UTR) with mature synthesis technology	Medium to high; preparation relies on back-splicing mechanism, requiring optimization of m6A modification or IRES elements to improve translation efficiency	Relatively high; complex structure (containing RdRp coding region, packaging signal and other elements), requiring optimization of RdRp sequence and delivery system
Safety	Relatively safe; no risk of integrating into the host genome; mild local inflammation, transient fever and other adverse reactions may occur, mostly transient	High; the natural circular structure has low immunogenicity and no risk of genome integration; good biocompatibility with low incidence of adverse reactions	Relatively safe; no risk of genome integration; potential immunogenicity needs precise regulation, and no serious adverse reactions have been found in current clinical trials

**Table 2:** Comparison of key performances of mainstream mRNA vaccine delivery systems.

Mainstream Delivery Systems	Delivery Efficiency	Toxicity	Scalability	Clinical Stage
Lipid Nanoparticles (LNP)	High; excellent endosomal escape ability, can efficiently deliver mRNA into host cells and promote antigen expression	Low; good biocompatibility, mild adverse reactions, and high safety verified by clinical practice	Highly feasible; mature large-scale preparation process, suitable for industrial mass production	Advanced; multiple mRNA vaccines based on LNP have been approved for marketing, and numerous candidates are in late-stage clinical trials
Dendritic Cells (DCs)	Moderate to high; inherent antigen-presenting ability, can specifically deliver mRNA and effectively induce cellular immune response	Low; autologous DCs are used in most cases, with low immunogenicity and few adverse reactions	Low; complex isolation, culture and modification processes, high cost, difficult to achieve large-scale production	Early to middle; mainly in preclinical research and early-phase clinical trials, few candidates enter late-phase trials
Protamine	Moderate; can bind to mRNA to form complexes and protect mRNA from degradation, but endosomal escape ability is weak	Low to moderate; generally safe, but may cause mild immune reactions in individual cases	Moderate; simple preparation process, but limited by relatively low delivery efficiency, large-scale application is restricted	Early; mostly in preclinical research and small-scale early-phase clinical trials, not yet approved for marketing
Exosomes	Moderate; good biocompatibility and targeting, can cross biological barriers, but loading capacity and delivery efficiency are lower than LNP	Low; derived from cells, good biocompatibility, low immunogenicity and toxic side effects	Low; complex separation and purification process, low yield, high cost, difficult to scale up	Early; mainly in preclinical research, a small number of studies enter early-phase clinical trials
Polymers (e.g., cationic polymers)	Moderate; can form stable complexes with mRNA to protect it from degradation, but endosomal escape ability varies with polymer type	Moderate; toxicity varies by polymer structure; some cationic polymers may cause cytotoxicity and inflammatory reactions	Moderate to high; relatively simple synthesis process, but some polymers have poor biocompatibility, limiting large-scale application	Early to middle; mostly in preclinical research and early-phase clinical trials, individual candidates enter middle-phase trials

**Table 3:** mRNA vaccines entering clinical trials.

Application Field	Vaccine Type/Target	Research Phase	Main Research Institutions	Key Clinical Trial Information
Prevention of Infectious Diseases	SARS-CoV-2	Marketed/Phase IV Clinical	Moderna, BioNTech/Pfizer, CSPC Pharmaceutical Group, etc.	mRNA-1273 (NCT04952402), BNT162b2 (NCT04368728), SYS6006 (NCT05492643)
	Influenza virus	Phase III Clinical	Moderna, Sanofi, Pfizer, etc.	mRNA-1010 (NCT05415462), mRNA-1083 (NCT06097273)
	Respiratory Syncytial Virus (RSV)	Marketed/Phase III Clinical	Moderna, Starna Therapeutics, Sanofi, etc.	mRNA-1345 (NCT06067230), STR-V003 (NCT06344975)
	Cytomegalovirus (CMV)	Phase II Clinical	Moderna	mRNA-1647 (NCT05085366)
	Rabies Virus (RV)	Phase I Clinical	CureVac, AmCan Biopharma, Jilin University Team, etc.	CV7202 (NCT03713086), LVRNA001, research on mRNA vaccine encoding full-length RABV-G by Jilin University
	Varicella-Zoster Virus (VZV)	Phase I/II Clinical	Moderna, BioNTech/Pfizer, etc.	mRNA-1468 (NCT05701800), BNT167 (NCT05703607)
	Monkeypox virus	Preclinical/Partially entering Phase I	Shenzhen Bay Laboratory, Tsinghua University, etc.	Study on combined vaccination of Mix-12 and MPX-EPs by Shenzhen Bay Laboratory (clinical trial number not registered yet)
Tumor Immunotherapy	Melanoma	Phase III Clinical	Moderna, BioNTech, etc.	mRNA-4157 (NCT05933577), BNT111 (NCT04526899)
	HPV-related tumors	Phase II Clinical	BioNTech, Renjing Biotechnology, etc.	BNT113 (NCT04534205), RG002 (NCT06273553)
	Prostate cancer	Phase I/II Clinical	CureVac	CV9103 (NCT00831467)
	EBV-positive solid tumors	Phase I Clinical	Chengdu Weishijin Biotechnology	WGc-043 (NCT05714748)
	Various solid tumors such as gastric cancer and intestinal cancer	Phase I/II Clinical	BioNTech, Likang Life Sciences, etc.	autogene cevumeran (BNT122) combination therapy research (NCT numbers correspond to multiple trials for different cancer types), LK101 injection (NCT numbers correspond to domestic studies)
	Squamous non-small cell lung cancer, head and neck squamous cell carcinoma, etc.	Phase I Clinical (domestic IND accepted)	Yunding Xinyao	EVM14 (domestic IND accepted, approved for clinical use by the US FDA, specific NCT number not yet announced)
Rare Disease Treatment	Methylmalonic Acidemia (MMA)	Phase I/II Clinical	Moderna	mRNA-3704 (NCT03810690)
	Cystic Fibrosis (CF)	Phase I/II Clinical	Translate Bio	MRT-5005 (specific NCT number not yet announced)

## Data Availability

All data presented are cited and publicly available. This article does not contain research data.
